# Application of AIIR algorithm for quality improvement and noise reduction in pediatric abdominal contrast-enhanced CT

**DOI:** 10.3389/fradi.2026.1803622

**Published:** 2026-05-15

**Authors:** Xie Jiazhi, Dai Dajian, Tang Shilong, Fan Xiao, Zhu Luyao, He Ling

**Affiliations:** 1 Department of Radiology, Children's Hospital Affiliated to Chongqing Medical University, Chongqing, China; 2 CT Business Unit, Shanghai United Imaging Healthcare Co., Ltd., Shanghai, China

**Keywords:** abdominal CT, children, contrast agent, deep learning iterative reconstruction, radiation dose

## Abstract

**Objective:**

To investigate the feasibility and value of the deep learning full model iterative algorithm (AIIR) in reducing radiation dose during contrast-enhanced whole abdominal CT scans in children.

**Methods:**

Data from 100 pediatric patients undergoing contrast-enhanced whole abdominal CT scans due to clinical indications were retrospectively collected. The patients were divided into a conventional group (*n* = 50,100 kVp, automatic current modulation 100 mAs) using hybrid iterative reconstruction (HIR) and an experimental group (*n* = 50,80 kVp, automatic current modulation 180 mAs) employing HIR and AIIR reconstruction. Subjective image quality scores (five-point scale evaluation), signal-to-noise ratio (SNR) and contrast-to-noise ratio (CNR) of major organs, contrast agent dosage, and radiation dose (CTDIvol, DLP, ED) were compared among the three groups during the arterial phase.

**Results:**

The experimental group exhibited significantly lower radiation dose compared to the conventional group (ED reduction by 23.61%, *P* < 0.001). In terms of image quality, subjective scores indicated superior performance of the AIIR group over both conventional HIR and experimental HIR groups (*P* < 0.05), with excellent inter-physician agreement (Kappa>0.70). The AIIR group demonstrated significantly higher SNR and CNR values for the liver, spleen, kidneys, gallbladder, pancreas, and abdominal aorta compared to the HIR groups (all *P* < 0.05).

**Conclusion:**

The AIIR algorithm achieves superior image quality at low dose levels (80 kVp) compared to conventional HIR and low-dose HIR protocols, demonstrating significant clinical value and potential for widespread application in pediatric abdominal imaging.

## 1 Introduction

Computed tomography (CT) is a crucial imaging modality for diagnosing pediatric abdominal diseases, but the ionizing radiation generated during scanning poses potential health risks to children. Pediatric tissues are in a developmental stage with active cell division, rendering them more susceptible to radiation-induced damage compared to adults. Studies have demonstrated that CT examinations during childhood are associated with an increased risk of subsequent intracranial tumors, leukemia, or lymphoma, particularly in children undergoing more than four CT scans (IRR 2.30,95%). Younger children (e.g., those with multiple scans) exhibit more pronounced cancer risks (1). Low-dose radiation may also elevate cancer risks, including hematologic malignancies (leukemia HR 1.074) and digestive system cancers (HR 1.285) (2). A European cohort study involving 948,000 individuals revealed that individuals who underwent CT scans before age 22 still face uncertain risks of hematologic malignancies even with lower dose levels (3). Therefore, minimizing radiation exposure while maintaining diagnostic image quality has become a critical challenge in pediatric imaging (4–6). Beyond radiation exposure, iodine-based contrast agents used in CT contrast-enhanced scans may also induce adverse reactions, particularly in children with immature renal function, where reducing contrast agent dosage holds significant clinical importance.

In recent years, researchers have adopted various methods to reduce CT radiation dose. Photon-counting CT (PCD CT) has demonstrated radiation dose reduction (median dose of 0.66 mSv vs. 1.39 mSv in conventional EID CT) while improving image signal-to-noise ratio (a 30% enhancement in renal regions) (7). Personalized protocols adjust abdominal CT planning based on patient risk, with optimized total risk reduction of 24% observed in Asian populations vs. only 5% in Hispanic populations. Optimization may involve dose escalation (in over 90% of cases), but clinical benefits must be weighed against radiation risks exceeding clinical thresholds by at least 400% (8). Combined optimization synchronizes radiation dose and contrast agent dose adjustments (reducing radiation by 26% or contrast agent by 16%) to achieve comparable image quality (9). Additionally, multiple iterative reconstruction techniques have been employed to significantly improve image quality and lesion detection rates while reducing radiation dose (10–12), though conventional iterative algorithms often struggle to strike an optimal balance between noise suppression and image texture fidelity (13). Deep learning-based full-model iterative algorithms (Artificial Intelligence Iterative Reconstruction, AIIR) integrate the physical modeling advantages of full-model iteration with deep learning's intelligent noise reduction capabilities, effectively minimizing image noise while preserving anatomical details and natural textures, thereby providing a novel technical approach for pediatric low-dose CT scanning (14–16). Currently, AIIR has demonstrated promising application potential in adult thoracic (17), abdominal (18), and cardiovascular (19) imaging, however systematic research on its application in pediatric abdominal contrast-enhanced CT scans remains insufficient.

This study aims to systematically evaluate the feasibility of the AIIR algorithm in reducing radiation dose and contrast agent dosage during pediatric abdominal contrast-enhanced CT examinations, and comprehensively assess the subjective and objective quality of reconstructed images, with the goal of providing safer and higher-quality pediatric abdominal CT scanning protocols for clinical practice. It is important to emphasize that the AIIR algorithm is a commercially available, fixed reconstruction software (United Imaging Healthcare) that was not modified or retrained for this study. Therefore, this investigation is a clinical validation study of an existing AI-based reconstruction algorithm, rather than a *de novo* AI model development study. Consequently, certain aspects such as internal model architecture, hyperparameter tuning, calibration, or uncertainty quantification are either not applicable or cannot be disclosed due to manufacturer's trade secrets.

## 2 Materials and methods

### 2.1 Study subjects

A retrospective study was conducted on 100 pediatric patients who underwent whole abdominal CT with contrast enhancement at Children's Hospital affiliated to Chongqing Medical University from February to August 2025 due to clinical indications. Participants were divided into a routine group (*n* = 50) and an experimental group (*n* = 50) based on scanning protocols: The routine group included 29 males and 21 females, aged 1.1–16.0 years (mean: 8.11 ± 4.51 years), with a body mass index (BMI) range of 11.34–28.34 kg/m^2^ (mean: 17.13 ± 4.07 kg/m^2^); The experimental group comprised 21 males and 29 females, aged 1.5–15.1 years (mean: 8.53 ± 4.07 years), with a BMI range of 11.49–27.90 kg/m^2^ (mean: 16.39 ± 3.15 kg/m^2^). Inclusion criteria were: (1) age range 1–16 years; (2) clinically indicated contrast-enhanced abdominal CT; (3) complete clinical and imaging data. Exclusion criteria were: (1) congenital or surgical absence of abdominal organs; (2) presence of abdominal metal implants causing significant artifacts; (3) renal dysfunction (eGFR < 60 mL/min/1.73 m2); (4) known allergy to iodine contrast agents. All enrolled patients had clinically justified indications for contrast-enhanced CT, and no patients were excluded for image quality reasons alone.This retrospective study was approved by the Ethics Committee of Children's Hospital Affiliated to Chongqing Medical University [Approval No.: (2025) Ethical Review (Clinical Research) Permit No.411]. All patient data were anonymized prior to analysis, and the requirement for informed consent was waived due to the retrospective design. The study was conducted in accordance with the Declaration of Helsinki. The ethics approval document is attached as a supplementary file.

### 2.2 Instruments and methods

All examinations were performed using the United Imaging uCT 968CT scanner. The scanning range extended from the apex of the diaphragm to the level of the lower border of the pubic symphysis. Scanning parameters were as follows: conventional group tube voltage 100 kVp with automatic tube current modulation (reference current 100 mAs), experimental group tube voltage 80 kVp with automatic tube current modulation (reference current 180 mAs). Both groups utilized a detector collimation width of 80 mm, rotation speed of 0.5 s/rot, and pitch of 0.90 mm. Contrast agent injection protocol: iohexol (350 μg/L) was administered as follows: conventional group for ages 1–7 years at a dose of 2.0 mL/kg, and for ages 8–16 years at a dose of 1.5–2.0 mL/kg; experimental group for ages 1–7 years at a dose of 1.5 mL/kg, and for ages 8–16 years at a dose of 1.0–1.2 mL/kg. Flow rate was adjusted according to body weight to 0.6–3.3 mL/s. All scans employed bolus tracking technology, with the monitoring plane positioned at the hepatic hilum level of the abdominal aorta, trigger threshold set at 200 HU, arterial phase scanning initiated after a 6-second delay, and venous phase scanning commenced after 60 s. Post-scanning image reconstruction was performed using hybrid iterative reconstruction (HIR) algorithm (Karl 3D, Shanghai United Imaging) in the conventional group, while the experimental group utilized AIIR and HIR algorithms with reconstruction layer thickness and spacing both set at 1 mm. All images were transmitted to the United Imaging post-processing workstation uOminspace. CT for image analysis. AIIR (Artificial Intelligence Iterative Reconstruction) is a reconstruction algorithm that integrates full model iteration with deep learning. It employs an end-to-end learning framework to impose hybrid-domain constraints in both the projection domain and image domain, utilizes high-quality data to train the network, and achieves precise separation of noise and anatomical signals. For detailed technical specifications, refer to the literature (14–16, 22–30). As the algorithm is proprietary, the manufacturer does not disclose the exact training dataset, network architecture details, or inference parameters. Interested readers are referred to the cited literature and the manufacturer's technical documentation for available information.

### 2.3 Image quality

#### 2.3.1 Subjective evaluation

Two physicians with over 8 years of pediatric imaging diagnostic experience independently assessed image quality using a double-blind method. The scoring criteria adopted a 5-point scale, with detailed scoring criteria as follows: 5 points: Excellent image quality with no significant artifacts, minimal noise points, and clear visualization of anatomical structures, details, and lesions; 4 points: Good image quality with minor artifacts and noise points, but anatomical structures and details remain clearly visible; 3 points: Average image quality with some artifacts and noise that do not affect diagnosis, identifiable anatomical structures, and relatively clear lesion visualization, meeting basic diagnostic requirements; 2 points: Poor image quality with prominent artifacts and noise, unclear anatomical structures, and unrecognizable details, impairing diagnosis; 1 point: Very poor image quality with obvious artifacts and noise, unrecognizable anatomical structures, and inability to diagnose; ≥3 points indicate diagnostic capability. Scoring ≥3 points was considered clinically significant. Disagreements between the two physicians were resolved through joint re-evaluation (20).

#### 2.3.2 Objective evaluation

**Figure 1:**
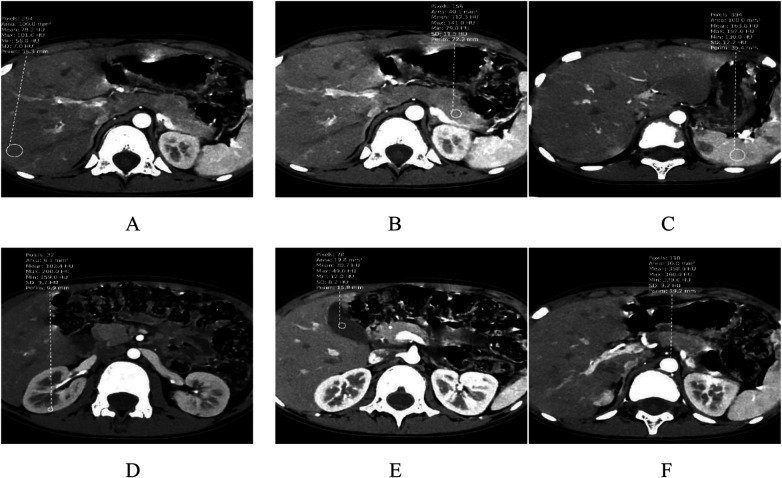
**(A)** liver ROI; **(B)** pancreas ROI; **(C)** spleen ROI; **(D)** kidney ROI; **(E)** gallbladder ROI; **(F)** abdominal aorta ROI.

Assessments were conducted by physicians with over 8 years of experience in pediatric imaging diagnosis. Circular regions of interest (ROI) were delineated on arterial-phase images at the following anatomical sites: liver (left middle posterior lobe approximately 100 mm^2^), pancreas (pancreatic tail approximately 40 mm^2^), spleen (splenic artery level in the central part of the spleen approximately 100 mm^2^), kidney (right renal cortex area at the posterior margin of the renal hilum approximately 8 mm^2^), gallbladder (central part of the gallbladder approximately 20 mm^2^), and abdominal aorta (level of the hepatic hilum approximately 30 mm^2^). The positive and negative errors of ROI delineation did not exceed 0.5 mm^2^, with care taken to avoid vascular structures and pathological areas. CT values and noise levels (standard deviation, SD) were measured, along with the CT values and SD of the erector spinae muscle at the same plane. The SD value of the erector spinae muscle was used as background noise to calculate the signal-to-noise ratio (SNR) and contrast-to-noise ratio (CNR). SNR = CT value of the ROI/SD of the ROI; CNR = (CT value of the ROI-CT value of the erector spinae muscle)/SD of the erector spinae muscle. The anatomical locations of the ROIs are illustrated in Figure 1. Measurements were performed three times for each anatomical site, with the mean value used as the final result.

#### 2.3.3 Diagnostic efficacy evaluation

Using clinical diagnostic results as the gold standard (positive for definite lesions, negative for no lesions or pending investigation), the mean scores of HIR and AIIR were used as test variables to plot receiver operating characteristic (ROC) curves, calculate the area under the curve (AUC) and 95% confidence interval (CI), and compare the differences between the two AUC values using the DeLong test.

### 2.4 Radiation dose assessment

Based on the CT scan dose report, the volume CT dose index (CTDIvol, mGy) and dose length product (DLP, mGy/cm) were recorded. The effective dose (ED, mSv) was calculated using the formula ED = DLP   ×   k, where k is the specific conversion factor for pediatric abdominal examinations, with corresponding k values selected according to age and body weight (range: 0.015–0.03 mSv·mGy⁻^1^·cm⁻^1^) (21).

### 2.5 Statistical analysis

Data analysis was performed using SPSS 26.0 software. Measurement data conforming to normal distribution were expressed as mean ± standard deviation ($\bar{x}$ ± s), while non-normal data were described using median and interquartile range. Between-group comparisons were conducted using the Mann–Whitney U test. Multivariate analyses for multiple group comparisons employed one-way ANOVA or Kruskal–Wallis test, with Bonferroni correction applied for multiple comparisons. The adjusted significance level was set at *P* < 0.05/m, where m denotes the number of comparisons. In addition to *P*-values, 95% confidence intervals (CI) and Cohen's d effect sizes were calculated. Cohen's d ≥ 0.2 was defined as small effect, 0.5 ≤ d ≤ 0.8 as moderate effect, and d ≥ 0.8 as large effect. Consistency in subjective scoring between two physicians was assessed using the Kappa test, with a Kappa value ≥0.75 indicating excellent agreement. All tests were two-sided, and a *P*-value <0.05 was considered statistically significant (adjusted thresholds were applied post-correction).

## 3 Results

### 3.1 General data

There were no statistically significant differences in age, gender, and BMI between the two groups of scanned children (*P* > 0.05). Specific data are shown in Table 1.

**Table 1:** Comparison of baseline characteristics between the control group and experimental group in pediatric patients (x ± s).

group	Gender (example)		Age (years)	weight （kg）	BMI (kg/m^2^)
	man	woman			
Routine (*n* = 50)	29	21	8.11 ± 4.51	28.38 ± 16.33	17.13 ± 4.07
Experiment (*n* = 50)	21	29	8.53 ± 4.07	29.20 ± 13.87	16.39 ± 3.15
*χ*^2^/Z/t price	1.094		−0.562	−0.648	−0.641
*P* price	0.296		0.574	0.517	0.521

### 3.2 Subjective evaluation

**Figure 2:**
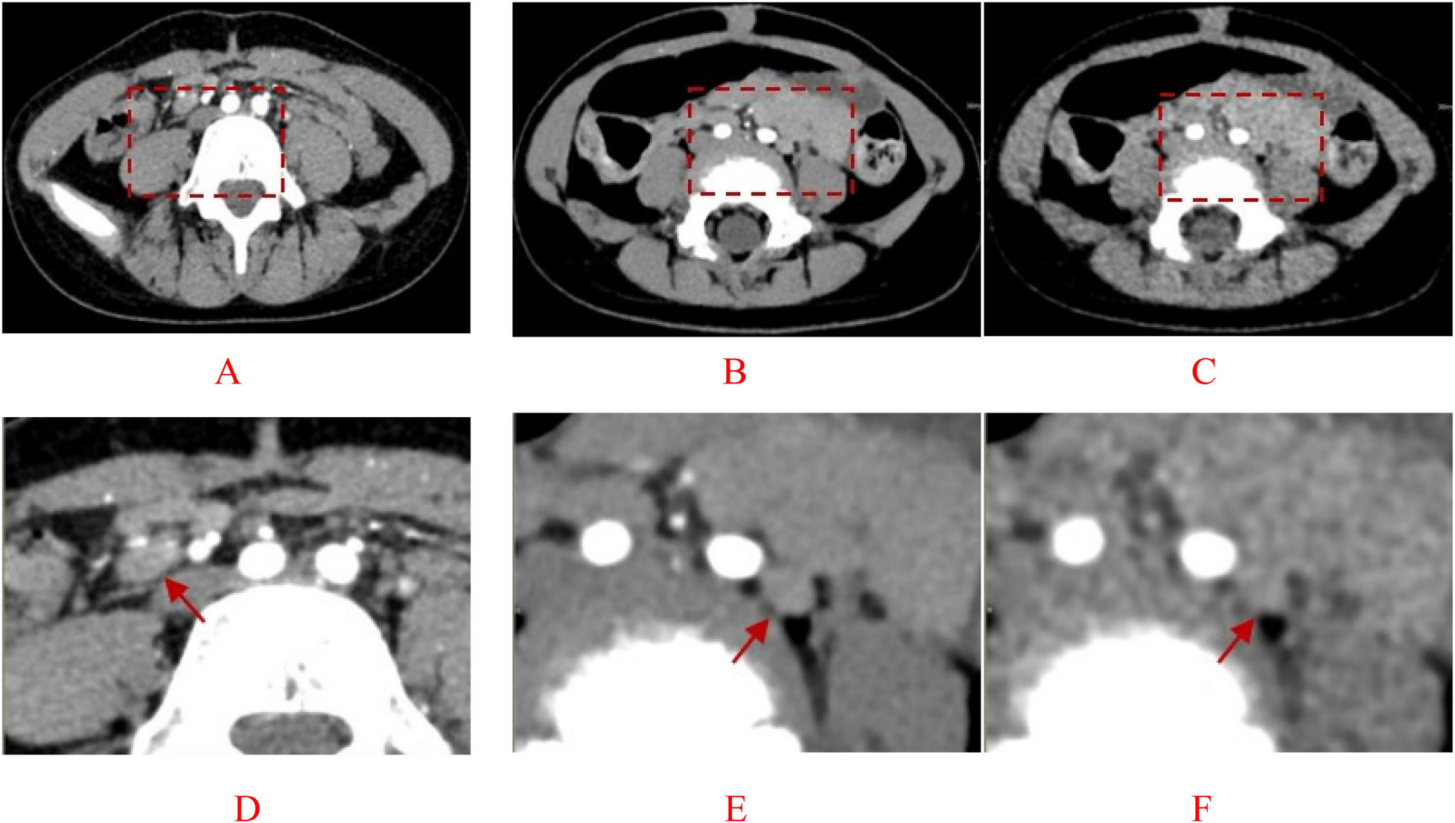
**(A)** conventional-dose HIR image (100 kVp) from a 13-year-old female with abdominal pain and lymphadenopathy. Enlarged lymph nodes (arrows) are visible but with moderate noise. **(B)** Low-dose AIIR image (80 kVp) from a 1-year-8-month-old female with dyspepsia and lymphadenopathy. Lymph node borders are sharper and background noise is markedly reduced compared to **(C)**. **(C)** Low-dose HIR image (80 kVp) from the same patient as **(B)**, showing increased image noise and reduced lesion conspicuity. **(D–F)**. Corresponding magnified views of the lesions in **(A–C)**, highlighting the superior detail preservation with AIIR.

**Figure 3:**
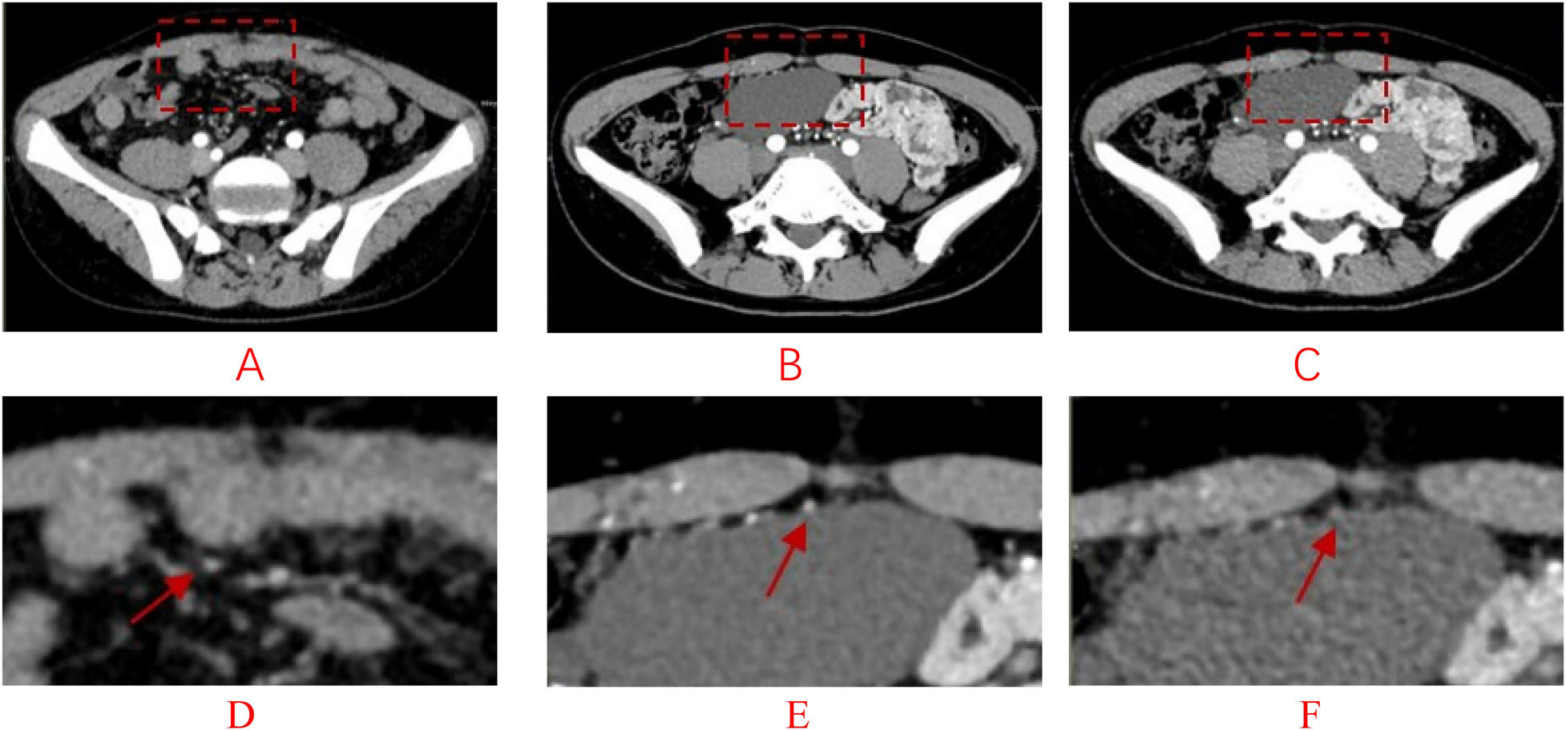
**(A)** conventional-dose HIR image (100 kVp) from a 12-year-old male patient with upper gastrointestinal bleeding, showing the ileal artery (arrow). Image noise is moderate but vascular delineation is acceptable. **(B)** Low-dose AIIR image (80 kVp) from a 15-year-old female patient with lymphangioma, demonstrating compression of the ileal artery (arrow) by the lymphangioma. The AIIR image exhibits markedly reduced noise and improved vessel wall definition compared to **(C)**. **(C)** Low-dose HIR image (80 kVp) from the same patient as **(B)**, showing increased background noise and slightly blurred arterial margins, reducing diagnostic confidence. **(D–F)**. Corresponding magnified views of the regions of interest in **(A–C)**, highlighting the superior noise suppression and edge preservation achieved with AIIR **(E)** compared to HIR **(C,F)**.

The two physicians demonstrated good consistency in scoring (Kappa = 0.71–1.00). The experimental AIIR group showed significantly higher subjective scores compared to the experimental HIR group (all *P* < 0.05), with enhanced clarity in multi-lesion tissues such as the intestines and urinary tract, as well as higher contrast between lesion sites and normal tissues. Representative images of lesion visualization are shown in Figure 2. Figure 3 illustrates the superiority of AIIR in vascular wall definition and noise suppression. Specific scoring results are presented in Table 2.

**Table 2:** Comparison of subjective evaluation results of images across groups.

group	Anatomical structure clarity		Artificial artifact control		noise control		Diagnostic confidence	
	Physician 1	Physician 2	Physician 1	Physician 2	Physician 1	Physician 2	Physician 1	Physician 2
convention HIR	4 (4, 4)	4 (4, 4)	4 (4, 4)	4 (4, 4)	5 (4, 5)	4 (4, 5)	4 (4, 5)	4 (4, 5)
Experiments HIR	3 (3, 4)	3 (3, 4)	4 (3, 4)	4 (3, 4)	4 (4, 4)	4 (4, 4)	4 (4, 4)	4 (4, 4)
Experiments AIIR	5 (4, 5)	5 (4, 5)	5 (4, 5)	5 (4, 5)	5 (5, 5)	5 (5, 5)	5 (5, 5)	5 (5, 5)
H price	61.23	62.92	56.07	59.55	66.04	65.02	65.31	66.12
P price	<0.001	<0.001	<0.001	<0.001	<0.001	<0.001	<0.001	<0.001

### 3.3 Objective evaluation

The experimental group's AIIR images demonstrated superior SD, SNR, and CNR compared to the experimental group's HIR, with statistically significant differences (Bonferroni correction *P* < 0.003). Compared to the conventional group's HIR, the experimental group's AIIR also showed improved SNR and CNR in most organs. Effect size analysis revealed that the experimental group's AIIR exhibited significant effects (Cohen's d ≥ 0.8) on SNR and CNR in the liver, pancreas, kidneys, gallbladder, and abdominal aorta compared to the experimental group's HIR, indicating clinically significant improvements in image quality (Table 3). The radiation dose and contrast agent usage for the two groups are compared in Table 4, and the detailed statistical results are presented in Table 5.

**Table 3:** Comparison of objective evaluation results of images across groups.

group	SD					
	hepar	pancreas	spleen	kidney	gall	aorta abdominalis
convention HIR	9.97 ± 2.08	11.38 ± 2.05	19.67 ± 7.76	9.62 ± 2.99	9.37 ± 2.27	14.73 ± 4.88
Experiments HIR	13.28 ± 2.29	14.93 ± 2.98	24.79 ± 9.23	12.89 ± 3.54	11.55 ± 2.33	18.17 ± 6.91
Experiments AIIR	7.74 ± 1.16	9.94 ± 2.39	24.17 ± 11.24	9.58 ± 3.29	7.16 ± 1.49	10.79 ± 5.13
F/H price	96.24	63.72	8.98	29.77	69.62	65.72
P price	<0.001	<0.001	<0.05	<0.001	<0.001	<0.001
d1*	2.92	1.86	0.06	0.98	2.20	1.17
d2*	1.18	0.65	0.45	0.01	1.17	0.80

group	SNR					
	hepar	pancreas	spleen	kidney	gall	aorta abdominalis
convention HIR	7.35 ± 1.80	10.13 ± 2.49	6.29 ± 3.11	20.62 ± 8.15	2.80 ± 1.26	27.68 ± 8.32
Experiments HIR	5.55 ± 1.15	8.58 ± 1.91	5.71 ± 2.296	17.09 ± 5.87	1.96 ± 0.99	26.83 ± 8.09
Experiments AIIR	9.29 ± 1.67	13.22 ± 3.22	6.49 ± 3.71	23.63 ± 7.31	2.81 ± 1.39	46.28 ± 12.39
F/H price	79.82	53.67	1.29	21.31	14.65	60.29
P price	<0.001	<0.001	0.5255	<0.001	<0.001	<0.001
d1	2.62	1.67	0.24	0.97	0.67	1.81
d2	1.11	1.08	0.06	0.39	0.01	1.71

group	CNR					
	hepar	pancreas	spleen	kidney	gall	aorta abdominalis
convention HIR	0.77 ± 1.25	5.40 ± 2.72	5.22 ± 3.56	13.11 ± 5.75	−4.32 ± 1.47	34.88 ± 10.36
Experiments HIR	0.68 ± 0.97	4.92 ± 1.86	4.97 ± 2.41	11.57 ± 3.70	−3.45 ± 0.97	30.83 ± 6.84
Experiments AIIR	0.99 ± 1.57	8.53 ± 3.24	8.25 ± 4.29	19.92 ± 6.60	−6.04 ± 1.57	52.80 ± 12.80
F/H price	2.56	37.29	22.27	44.68	59.314	66.18
P price	0.278	<0.001	<0.001	<0.001	<0.001	<0.001
d1	0.23	1.29	0.86	1.51	1.84	1.94
d2	0.16	1.03	0.72	1.07	1.06	1.56

* d1: Cohen's d effect size for experimental AIIR vs. experimental HIR.

* d2: Cohen's d effect size for experimental AIIR vs. conventional HIR.

**Table 4:** Comparison of radiation dose and contrast agent usage between the conventional group and experimental group (x ± s).

group	CTDIvol (mGy)	DLP (mGy·cm)	ED (mSv)	Dose of contrast agent (mL)	Contrast agent flow rate (mL/s)
convention	4.06 ± 0.96	186.10 ± 72.96	3.60 ± 0.95	44.42 ± 17.55	2.05 ± 0.83
Experiments	3.15 ± 0.77	146.92 ± 51.83	2.75 ± 0.62	41.32 ± 16.28	2.03 ± 0.80
Z price	−4.922	−2.647	−5.095	−1.012	−0.149
P price	<0.001	0.0081	<0.001	0.3116	0.8819

**Table 5:** Summary of key image quality and radiation dose parameters across groups.

Parameter	Conventional HIR (100 kVp) (*n* = 50)	Experimental HIR (80 kVp) (*n* = 50)	Experimental AIIR (80 kVp) (*n* = 50)
Subjective diagnostic confidence score (median, IQR)	4 (4–5)	4 (4–4)	5 (5–5)
Liver SNR	7.35 ± 1.80	5.55 ± 1.15	9.29 ± 1.67
Liver CNR	0.77 ± 1.25	0.68 ± 0.97	0.99 ± 1.57
Abdominal aorta SNR	27.68 ± 8.32	26.83 ± 8.09	46.28 ± 12.39
CTDIvol (mGy)	4.06 ± 0.96	3.15 ± 0.77	—
Effective dose ED (mSv)	3.60 ± 0.95	2.75 ± 0.62	—

Data are presented as mean ± standard deviation or median (interquartile range). Radiation dose parameters for the experimental AIIR group are identical to those of the experimental HIR group (same scanning protocol) and are therefore not repeated. All comparisons between the experimental AIIR group and the experimental HIR group showed statistically significant differences (Bonferroni-corrected *P* < 0.003) with large effect sizes (Cohen's d ≥ 0.8). Subjective scores are derived from Table 2, objective parameters from Table 3, and radiation doses from Table 4.

### 3.4 Diagnostic efficacy evaluation

**Figure 4:**
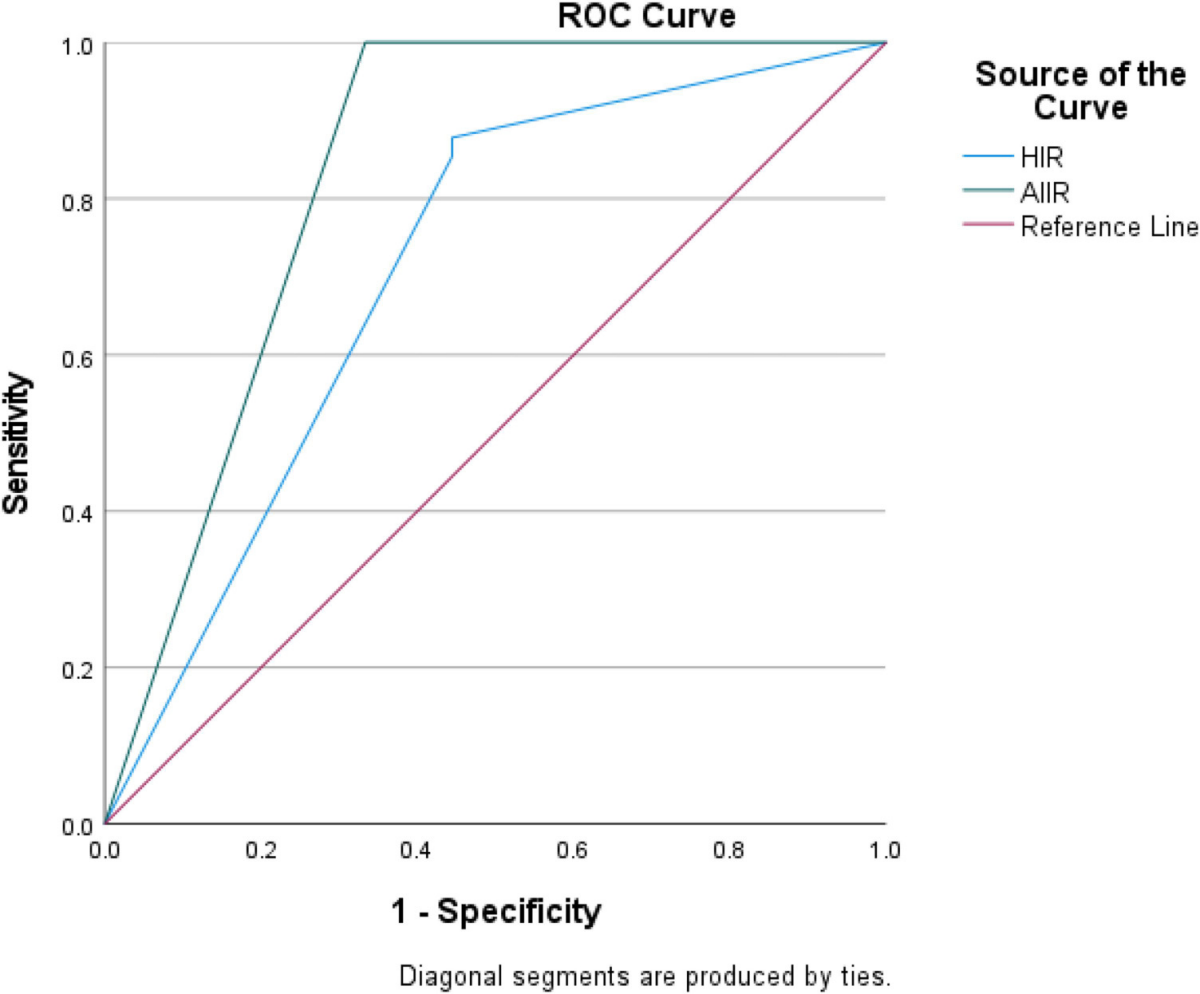
Receiver operating characteristic (ROC) curve of diagnostic efficacy for HIR and AIIR.

Using clinical diagnostic results as the gold standard (positive for definite lesions, negative for no lesions or pending investigation), ROC curve analysis (Figure 4) demonstrated that the AIIR score had an AUC of 0.833 (95% CI: 0.670–0.997) for diagnosis, while the HIR score had an AUC of 0.711 (95% CI: 0.528–0.895). DeLong's test revealed no statistically significant difference between the two scores (*P* = 0.365), indicating that although AIIR exhibited numerically higher diagnostic efficacy in this study sample, it did not reach statistical significance.

### 3.5 Radiation dose and contrast agent administration

The radiation dose parameters in the arterial phase CT scan of the experimental group were significantly lower than those in the conventional group (all *P* values <0.05), with CTDIvol, DLP, and ED reduced by 22.36%,21.05%, and 23.61%, respectively. No significant differences were observed in contrast agent dosage or flow rate.

## 4 Discussion

This study compared the reconstruction performance of conventional dose mixed iterative reconstruction (HIR) with low-dose HIR and AIIR, demonstrating that the AIIR algorithm significantly improves image quality and reduces noise in pediatric abdominal contrast-enhanced CT while achieving effective radiation dose reduction. The results showed that in the experimental group, when the tube voltage was reduced to 80 kVp and the reference tube current was appropriately increased to 180 mAs, the effective dose (ED) decreased by 23.61% compared to the conventional group. AIIR reconstructed images outperformed HIR images under the same dose conditions in subjective evaluation, signal-to-noise ratio (SNR), and contrast-to-noise ratio (CNR), with some metrics even surpassing those of conventional dose HIR images. These findings indicate that AIIR maintains or enhances overall diagnostic image quality while reducing radiation dose.

The advantages of AIIR primarily stem from its integration of full-model iterative physical accuracy and deep learning-based adaptive denoising capabilities (22, 23). While traditional iterative algorithms can reduce noise, they are prone to causing image texture distortion or “plasticity,” which impairs the identification of fine structures (24). In contrast, AIIR, trained on extensive high-quality image data, achieves more precise differentiation between true anatomical signals and noise, effectively suppressing noise while preserving natural image appearance and detail clarity (25). This is particularly beneficial for displaying multiple structures and lesions in pediatric abdominal imaging, thereby enhancing diagnostic confidence (26, 27). The advanced performance of AIIR is further demonstrated by its end-to-end learning framework (28) and hybrid-domain constraints (29), which significantly improve reconstruction speed and accuracy, making it especially suitable for rapid imaging needs in pediatric patients (26, 30).

Additionally, although the contrast agent dosage in the experimental group was slightly reduced in this study, no statistically significant difference was observed. This suggests that the combination of AIIR and low tube voltage protocols may provide a viable approach for further exploration of “dual-low” (low radiation, low contrast agent) scanning while maintaining enhancement efficacy. Given that pediatric patients have immature organ functions, dose reduction of contrast agents offers clear clinical benefits. Future research could focus on optimizing injection protocols to achieve dual safety control of radiation and contrast agent exposure.

This study further evaluated the diagnostic efficacy of AIIR and HIR for real lesions using clinical diagnostic results as the gold standard. ROC analysis revealed that the AUC of AIIR was 0.833, while that of HIR was 0.711, but the difference was not statistically significant (*P* = 0.365). This result is not entirely consistent with the conclusion that AIIR significantly outperforms HIR in image quality evaluation (SNR, CNR, subjective scoring). Possible reasons include: ① A high proportion of positive cases (80%) and only 10 negative cases in the study sample, resulting in insufficient statistical power to detect true differences in AUC; ② Diagnostic results derived from comprehensive clinical diagnoses, with some cases lacking pathological gold standards, potentially introducing classification bias; ③ The image quality advantage of AIIR primarily lies in noise suppression and detail visualization, but its improvement in lesion detection may require larger sample sizes to demonstrate.

Our findings are consistent with previous studies that demonstrated improved image quality with deep learning-based iterative reconstruction in adult populations (17–19). However, this study specifically focuses on pediatric abdominal imaging, where radiation sensitivity is higher and image quality demands are more stringent. Compared with hybrid iterative reconstruction, the AIIR algorithm achieved a 23.6% reduction in effective dose while maintaining or improving SNR and CNR, which aligns with the ALARA principle. Notably, the diagnostic efficacy (AUC) did not reach statistical significance, likely due to the imbalance in case–control distribution (80% positive cases) and limited sample size. Similar observations have been reported in other validation studies of commercial AI reconstruction tools, where image quality improvements do not always translate into immediately measurable diagnostic accuracy gains without adequately powered studies (26, 27). Future prospective studies with balanced disease spectra and pathological gold standards are needed to clarify the diagnostic added value of AIIR.

This study has the following limitations: First, regarding sample representativeness, the study was designed as a single-center retrospective study with relatively limited sample size and no subgroup analysis based on age or body weight. The generalizability of the conclusions requires validation through multicenter, large-sample studies. Importantly, because the AIIR algorithm is a fixed commercial product that was not retrained or fine-tuned using our data, there is no risk of data leakage between development and evaluation datasets. The algorithm's performance in this study reflects its real-world generalizability to a new patient population, although external validation in other centers remains desirable. Second, in terms of diagnostic efficacy, although the ROC analysis used clinical diagnosis as the gold standard, the excessively high proportion of positive cases may compromise statistical power, and some diagnoses lacked pathological confirmation. Third, concerning the scope of the study, the focus was primarily on arterial phase imaging, while the AIIR reconstruction performance in venous phase and equilibrium phase warrants further investigation. Furthermore, as a clinical validation of a commercial algorithm, this study does not address the internal workings of the AI model; such information remains proprietary to the manufacturer. Nevertheless, our results provide real-world evidence supporting the use of AIIR in pediatric low-dose CT protocols.

In conclusion, the AIIR algorithm demonstrates significant potential to reduce radiation dose exposure in pediatric abdominal contrast-enhanced CT scans while maintaining superior image quality, exhibiting high clinical application value and broad implementation prospects. Future studies should expand sample cohorts, establish multicenter collaborations, and implement longitudinal follow-up studies to further validate AIIR's diagnostic efficacy across various diseases and prognostic assessments. These efforts will propel pediatric imaging examinations toward safer and more accurate diagnostic approaches.

## Data Availability

The original contributions presented in the study are included in the article/supplementary material, further inquiries can be directed to the corresponding author, Ling He: heling508@sina.com.
